# Identification of a New Pathogenicity Island Within the Large pAH187_270 Plasmid Involved in *Bacillus cereus* Virulence

**DOI:** 10.3389/fcimb.2021.788757

**Published:** 2022-01-20

**Authors:** Rozenn Dervyn, Devon W. Kavanaugh, Delphine Cormontagne, Benjamin Glasset, Nalini Ramarao

**Affiliations:** Université Paris-Saclay, INRAE, Micalis Institute, Jouy-en-Josas, France

**Keywords:** *Bacillus cereus*, clinical infection, virulence, mega-plasmid, pathogenicity island

## Abstract

**Objectives:**

*Bacillus cereus* is responsible for food poisoning and rare but severe clinical infections. The pathogenicity of *B. cereus* strains varies from harmless to lethal strains. The objective of this study was to characterize three *B. cereus* isolates isolated from the same patient and identify their virulence potentials.

**Methods:**

Three isolates of *B. cereus* were isolated from various blood samples from a patient who developed sepsis following a central venous catheter infection. The three isolates were compared by WGS, genotyping and SNP analysis. Furthermore, the isolates were compared by phenotypical analysis including bacterial growth, morphology, germination efficacy, toxin production, antibiotic susceptibility and virulence in an insect model of infection.

**Results:**

According to WGS and genotyping, the 3 isolates were shown to be identical strains. However, the last recovered strain had lost the mega pAH187_270 plasmid. This last strain showed different phenotypes compared to the first isolated strain, such as germination delay, different antibiotic susceptibility and a decreased virulence capacity towards insects. A 50- kbp region of pAH187_270 plasmid was involved in the virulence potential and could thus be defined as a new pathogenicity island of *B. cereus*.

**Conclusions:**

These new findings help in the understanding of *B. cereus* pathogenic potential and complexity and provide further hints into the role of large plasmids in the virulence of *B. cereus* strains. This may provide tools for a better assessment of the risks associated with *B. cereus* hospital contamination to improve hygiene procedure and patient health.

## Introduction

*Bacillus cereus* is a Gram positive, spore-forming bacterium found in nearly all environments. The pathogenic potential of the strains of *B. cereus* ranges from beneficial to benign to pathogenic (Stenfors Arnesen et al., 2008). *B. cereus* is the 3rd most frequent bacterial agent responsible for food-borne outbreaks in Europe (Journal, 2009). In addition, infection with *B. cereus* is associated with non-gastrointestinal diseases and can potentially result in pneumonia, septicaemia, endocarditis, and meningitis, with immunocompromised individuals and neonates being particularly susceptible (Bottone, 2010; Cormontagne et al., 2021). *B. cereus* is able to persist in the environment over long periods and can cause recurrent nosocomial infections (Glasset et al., 2018).

Among the most widely studied toxins of *B. cereus* are those related to the diarrheal syndrome, which include non-haemolytic enterotoxin (Nhe), Haemolysin BL (Hbl), and Cytotoxins K (CytK1 and CytK2), all of which are pore-forming toxins (Granum and Lund, 1997; Fagerlund et al., 2008; Guinebretiere et al., 2013; Ramarao and Sanchis, 2013). Additionally, enzymatic proteins have been identified, which contribute to *B. cereus* toxicity, such as InhAs and CwpFM (Haydar et al., 2018; Tran et al., 2020). Furthermore, the causative agent of emesis, the dodecadepsipeptide cereulide, is restricted to strains carrying pXO-1-like megaplasmids (Carlin et al., 2006). Although these factors are involved in *B. cereus* pathogenicity, the differentiation of pathogenic from non-pathogenic strains has proven difficult, even with molecular methods (Ramarao et al., 2020).

We have recently identified a subset of genes, not previously associated with virulence, which presence helps to discern clinical from harmless strains (Kavanaugh et al., 2021). Considering the difficulty in discerning strains as well as the time required for phenotypic tests, it is anticipated that future methodologies will focus on risk-orientated differential diagnostics, through inclusion of methods for detection of toxins, toxin genes and markers of virulence (Fricker et al., 2007).

In the present study, 3 strains were isolated from the same patient over an 87-day period. The first two strains were virtually identical with the third having lost a mega plasmid that contains several of the recently identified biomarkers. This last isolated strain had a decreased capacity to germinate and to induce virulence in an insect model, strongly suggesting that the pAH187_270 plasmid promotes virulence.

## Materials and Methods

### Bacterial Strains and Growth Conditions

A 63-year-old male patient was admitted at a French University Hospital with Crohn’s disease and chronic renal failure. The patient further developed sepsis and a central venous catheter infection. *B. cereus* isolates were isolated from various blood samples from this patient.

The patient underwent intensive rounds of antibiotic treatment including amoxicillin, ciprofloxacin (21 days), gentamycin (3 days), imipenem (18 days) followed by ciprofloxacin and vancomycin (10 days) (Glasset et al., 2018). Three positive blood cultures yielding *B. cereus* isolates (12CEB42BAC_S94, 12CEB40BAC_S20, 12CEB43BAC_S95, further named S94, S20 and S95, respectively) were obtained during a span of 87 days. The first sample (S94) was collected before the patient received antibiotic treatments, the second sample (S20) was collected after 23 days of treatment, and the last sample (S95) was collected 64 days after the previous one. The isolated strains were confirmed as being *B. cereus* by MALDI-TOF and no other bacteria were isolated in the blood cultures. The patient was later released from hospital, presenting no further signs of infection.

### DNA Extraction, Genome Sequencing, and Assembly

The bacterial strains were grown overnight in BHI medium at 37°C, 200 rpm until mid-exponential growth phase and bacteria were pelleted. Total DNA was extracted as previously described (Cadot et al., 2010; Kavanaugh et al., 2021) and quantified with the Qubit^®^ Fluorimeter. DNA concentrations were adjusted to 30 ng/ml and sequenced by the MiSeq Illumina platform hosted at the Pasteur Institute, giving 2x150 bp paired-end reads.

Sequencing analysis was performed as previously described (Kavanaugh et al., 2021) and included quality control using FastQC and MultiQC, *de novo* assembly of draft genomes with SPAdes version 3.13.0. Raw WGS data are accessible through https://www.ebi.ac.uk/ena/ (accession number PRJEB46455).

The genome sequences of the 3 strains were aligned against the pAH187_270 plasmid from the strain *B. cereus* AH187 using NUCmer for sequence alignment and MUMmer/MUMmerplot for visualisation/coverage plots. Further analysis was performed using the Proksee server (https://beta.proksee.ca/) to create circular alignments of reads to the reference plasmid.

### Genotyping, Genome Annotation, and SNPs Analysis

MLST was determined for the 3 strains using the online MLST tool available from the Centre for Genomic Epidemiology (Larsen et al., 2012). Genome annotation was carried out using the Prokka automatic annotation tool v [1.13] (Seemann, 2014). Core and accessory genomes were determined by Roary with default settings, with the core genome defined as genes present in at least 3 of 4 samples, including *B. cereus* AH187 as reference strain.

SNPs were identified using Snippy (Seemann, 2014), which infers polymorphisms at the nucleotide level by aligning the sequencing reads against the reference plasmid pAH187_270.

### Molecular Analysis

M13 sequence-based polymerase chain reaction (M13-PCR), derived from a RAPD technique, allows differentiating between various strain patterns. M13 typing was performed as described (Glasset et al., 2016). The DNA profiles were analyzed with BioNumerics 7.1 software (Applied Maths). The software compared the DNA profiles and clustered the strains according to their similarity.

The toxin gene profiles were identified by assessing the presence of the *cytK-1, cytK-2, HBLA, HBLC, HBLD, NHEA, NHEB, NHEC, hlyII* and *ces* genes by PCR using specific primers (Glasset et al., 2016). The strains were then clustered into genetic signatures (GS) according to their different combinations of presence/absence patterns (Glasset et al., 2021).

The strains were affiliated to one of the seven known phylogenetic groups according to the partial sequencing of the *panC* gene (Guinebretière et al., 2008). The production of the enterotoxins NHE and HBL was tested with the immunological tests BCET-RPLA Toxin Detection (Oxoïd) and Tecra (BDE VIA, 3M-Tecra) kits, respectively (Guinebretière et al., 2002).

### Plasmid Curing

Plasmid-curing was tempted to determine the influence of the 270 kb pAH187 plasmid on strain characteristics. Plasmid-curing was investigated through culturing the strains at increased temperature (41°C and 43°C), addition of antibiotics (ampicillin – 50µg/ml or novobiocin - 2ug/ml), or ethidium bromide (15, 100, 125 µg/ml) during repeated or prolonged culture between 5-10 days.

### Pathogenicity Island (PAI) Deletion on the pAH187 Plasmid

Knock-out of the region corresponding to the PAI on the pAH187 plasmid carried by the S94 strain was accomplished by double-cross over region substitution. Briefly, using the available sequencing information of the pAH187 plasmid (NC_011655), 1 kb regions upstream (region 130550 to 131543) and downstream (region 174739 to 175734) of the identified PAI were synthesized by the Genecust company (Boynes, France). A spectinomycin-resistance cassette was obtained from pAT28 (Trieu-Cuot et al., 1990). The two fragments upstream and downstream of the PAI were cloned at each side of the cassette into the pAT113 vector (Trieu-Cuot et al., 1991) by the Genecust company. The constructed plasmid was named as pAT113 Δpai-pAH187 and transformed into chemically competent *E. coli* ET12567 and then by conjugation into *B. cereus* S94 strain (Trieu-Cuot et al., 1987; Huys et al., 2004). Briefly, the donor strain and receptor strains were mixed at ratio 14:1 on a sterile membrane filter with a pore size of 0.05 µm (VMWP02500 Milipore) deposited on BHI agar plates for 18 h at 37°C. After mating, bacteria were resuspended from filter with 1.5 mL of colicin solution (Hill and Holland, 1967). The transconjugates were selected on BHI agar plates supplemented with 300 µg/mL of spectinomycin at 37°C. The deletion of the PAI by double recombination event was verified by PCR using primers located upstream and downstream of the cloned region. The corresponding mutant was named S94 Δpai. Due to the size of the entire PAI, it was technically not possible to obtain a complemented strain.

### Growth and Morphology

All strains were inoculated into BHI broth and grown at 37°C, 200 rpm. Bacterial growth was followed by measuring the OD at 600 nm at regular intervals.

To determine cellular morphology and size, bacteria were observed on BHI plates. The size of 25 colonies per plate was measured with a graduated scale. Alternatively, bacteria harvested at the end of exponential growth phase were observed under an AxioObserver. ZI Zeiss inverted microscope.

### Sporulation/Germination

The sporulation efficiency of the strains was determined in HCT plus 0.3% glucose as previously described (Ramarao and Lereclus, 2005). For germination assays, the spores were incubated in BHI medium for 55 min. Samples were taken at 0, 7 min, 16 min, 25 min, 40 min, 55 min. The number of remaining spores was determined as heat-resistant (85°C for 15 min) CFU on BHI plates and normalized to the initial spore value at T0.

### Insect Experiments

Spores were injected at various concentrations between the second and third body segment from the rear of 10 last instar *Galleria mellonella* larvae as previously described (Buisson et al., 2019). The mortality rate was measured after 24 h of infection at 37°C.

### Antibiotic Susceptibility

The Minimum Inhibitory Concentrations (MICs) of selected antimicrobial agents were measured by using concentration gradient strips (Etest^®^, BioMerieux) (Glasset et al., 2018). The following agents were tested: ampicillin^$^, cefotaxime, imipenem^$^, vancomycin^$^, gentamicin^$^, rifampicin^$^, tetracycline^$^, ciprofloxacin^$^, chloramphenicol^$^, azithromycin, sulfamethoxazole/trimethoprim^$^ and clindamycin^$^. Due to scarce availability of interpretative criteria in the literature, clinical breakpoints were used when available (^$^) (Wayne and Clinical and Laboratory Standards Institute (CLSI), 2010).

## Results

### Comparison of the Three Isolates

Three *B. cereus* isolates were isolated from three different blood cultures of the same patient within a period of 87 days and following intensive antibiotic treatments. No other bacteria were isolated from the blood cultures.

**Figure 1:**
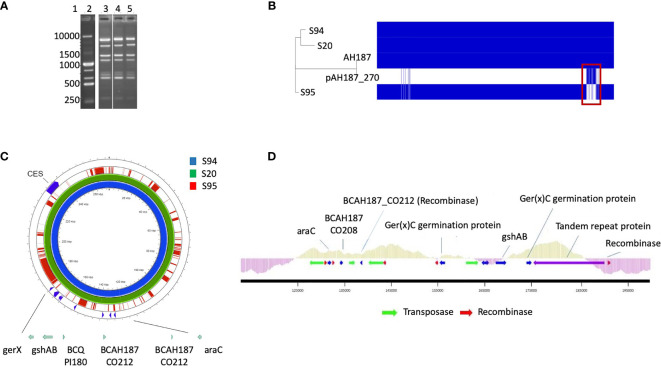
**(A)** M13-PCR fingerprint patterns, Lane 1: 1 kb DNA ladder. Lane 2: reference strain *B cereus* ATCC14579. Lane 3 to 5: S94, S20, S95 *B cereus* strains. **(B)** Visualization of core and accessory genomes of patient isolates S94, S20, S95, along with reference strain *B cereus* AH187 and mega-plasmid pAH187_270, using RAxML-generated phylogenetic tree and presence/absence table generated by Roary. Red box highlights absence of pAH187_270 genes in strain S95. **(C)** Proksee visualization of strains S94, S20, S95 aligned to the plasmid pAH187_270 nucleotide sequences. Key biomarkers are identified within the plasmid (green arrows), as well as the *ces* encoding gene. **(D)** Overview of the PAI. The plasmid region from 125,000 to 192,000bp of pAH187_270 contains all of the plasmid-based biomarkers (blue arrows) and several transposases/recombinases (green and red arrows, respectively). The average GC content is indicated above the genes within the PAI (light green) and in the surrounding regions of the plasmid (purple).

The M13 profiles of the 3 isolates were compared and were highly similar for the 3 isolates, suggesting that the 3 isolates are identical or very similar strains (**Figure 1A**).

In order to further compare the isolates, their genomes were sequenced. Mean size of the draft genomes was 5,533,542bp (range 5318760 [S95] - 5643975 [S94]). Mean GC % was 35.3%. *In silico* MLST analysis determined each strain to belong to the same MLST type 26.

The strains were further characterized for the presence of 10 genes implicated in virulence, the production of Nhe and the phylogenic group. The three isolates possess the *nhe* gene and were high Nhe producers. The *hbl* and *cytK* genes were absent in all isolates. The 3 isolates belong to phylogenetic group III. However, the *ces* gene was present in the two first isolates (S94 and S20) and absent in the last isolate (S95).

The patient received an intensive antibiotic treatment. To assess the potential impact of this treatment on the antibiotic resistance of the strains, the CMI against major antibiotic were measures for the first S94 and the last S95 isolated strains. The two isolates were resistant to ampicillin and cefotaxime. The strains were sensitive to the antibiotics that were administered to the patient. Strikingly, the 94 strain was susceptible to rifampicin whereas S95 strain displayed resistance to rifampicin (**Table 1**).

**Table 1:** MIC results (Etest method) for S94 and S95 *B*. *cereus* strains for 12 antibiotics.

Key	Ampicillin	Cefotaxime	Imipenem	Vancomycin	Gentamicin	Rifampicin	Tetracyclin	Ciprofloxacin	Chloramphenicol	Azithromycin	Sulf + Trimethoprime	Clindamycin
Recommandation CLSI	S< 0,25 - R >0,5	S < 8 - R > 64	S < 4 - R > 16	S < 4	S < 4 - R > 16	S < 1 - R > 4	S < 4 - R > 16	S < 1 - R > 4	S < 8 - R > 32	?	S < 2/38 - R > 4/76	S < 0,5 - R > 4
S94	1,5	64	0,047	1	0,094	**0,19**	0,38	0,047	3	0,19	0,25	0,19
S95	3	64	0,047	3	0,19	**6**	0,19	0,094	1,5	0,125	0,75	0,125

### Identification of a Missing Plasmid in the Last Isolated Strain

Mash analysis of the 3 strains identified the closest strain to S94 and S20 to be AH187 with an average nucleotide identity of 99.9%. The AH187 strain possesses the pAH_187- 270 kb mega-plasmid. Analysis of the core and accessory genomes reveals that nearly all variability among the 3 patient strains results from the accessory genes located on this mega-plasmid (**Figure 1B**).

To further assess the differences among the patient-isolated strains, snps were examined *via* Snippy using the plasmid, pAH187_270, as reference. A significant amount of variation is evident when comparing the S95 patient strain against the mega-plasmid, pAH187_270 (**Table 2**).

**Table 2:** Snippy SNP analysis of patient strains against the plasmid pAH187_270 as reference.

Variants	pAH187_270 plasmid		
Strain	S94	S20	S95
Complex	14	14	303
MNP	1	1	10
SNP	60	61	833
Insertion	0	0	2
Deletion	1	1	1
Total	76	77	1149

Consistently, the pAH187_270 plasmid sequence resulted in nearly 100% coverage for S20 and S94 strains. By contrast, the S95 strain did not demonstrate significant alignment to the pAH187_270 plasmid (**Figure 1C**).

Altogether, the 3 isolates have an almost identical chromosome but differed by the absence of the pAH187_270 plasmid in the S95 isolate compared to the S94 and S20 isolates.

### Identification and Reconstruction of a Pathogenicity Island

A closer look at the pAH187_270 plasmid revealed that some of the biomarkers previously identified as characteristic of clinical strains (Kavanaugh et al., 2021) were present on this plasmid (i.e., araC, gshAB, BCQ_PI180) (**Figure 1C**).

We confirmed by WGS that S20 and S94 carried these biomarkers, whereas the S95 strain did not. In addition, S94 and S20 carried the *ces* gene located in the pAH187_270 plasmid whereas S95 did not (not shown).

Based on the NUCmer alignment results, the localisation of the plasmid-based biomarkers of the S94 strain was determined. These biomarkers were located within a 43 kb span of the pAH187_270 plasmid, being distant from the CES operon for cereulide production (**Figure 1C**). We named this region the pathogenicity island (PAI). At first glance, it was observed that the PAI is flanked by resolvases and transposons indicating that they may have been obtained during horizontal gene transfer (**Figure 1D**). Further investigation of the plasmid pAH187 and the PAI revealed typical features used in defining PAIs. First, there is an abundance of direct repeats found in the plasmid. When a higher upper limit is set for maximum size (10,000 bp), 57 repeats of varying size, with 10 repeats of 396 bp clustered in the 175,466 to 189,105 region. However, with a lower limit of 200 bp, 20 hits are detected, with repeats detected on either side of the PAI at approx. 135,000 bp and 185,000 bp. Interestingly, pAH187 possesses a large inverted repeat 2,654 bp long, located 127,621-130,299 and 163,537-160,858, (99% match) found within the PAI. Furthermore, within the PAI, there is an increased presence of resolvases and transposases, which diminish in prevalence immediately outside the PAI. These are believed to play a role in the mobility of the DNA elements and facilitate their transfer and integration. Terminating one end of the PAI, a region, which spans from 175,000bp to 190,000 bp contains numerous tandem repeats, and finally terminates with a Holliday junction resolvase, further confirming the presence of the PAI.

Lastly, PAIs are often accompanied by changes in GC content in the inserted region, and this may be attributed to the DNA arriving from a different species through horizontal gene transfer. The GC content of the PAI was above average, compared to the immediately surrounding regions. It is to note that *B. cereus* is a low-GC-content bacteria and an increase in the GC content may thus indicate an integration of the PAI from a different species. However, as the difference in GC content is not a stark change, it may also represent an insertion or even a fusion event, as *B. cereus* megaplasmids (>100kb) have previously been hypothesized to result from fusion of smaller plasmids given the presence of multiple minireplicons (Zheng et al., 2013).

Several genes are present in the PAI: araC, BCAH187_C0208, BCAH187_C0212 being found at one end, BCN_P218 and BCAH187_C0212_2 towards the middle, and BCQ_PI180, BCQ_PI181, gshAB, 3 germination proteins encoded (including Ger(x)C), and a single protein (DUF11 domain-containing protein) being found on the opposing end of the pathogenicity island (**Table 3**).

**Table 3:** PAI genes with gene position (on the reference genome pAH187_270 - NC_011655.1), putative function and occurrence (%) in the strain collection.

Marker name	BCQ_PI180	gshAB	BCQ_PI181	gerX
**Gene name**	BCAH187_RS28565	BCAH187_C0244	BCAH187_RS28570	BCAH187_RS28600
**Gene position**	164163 | 164519 (plasmidic)	167109 | 169376 (plasmidic)	164642 | 165757 (plasmidic)	171639 | 172793 (plasmidic)
**Gene length**	357 nt	2268 nt	1116 nt	1155 nt
**Potential function**	helix-turn-helix transcriptional regulator	bifunctional glutamate–cysteine ligase GshA/glutathione synthetase GshB	S-(hydroxymethyl)glutathione dehydrogenase/class III alcohol dehydrogenase	Ger(x)C germination protein
**% in non clinical strains**	9	9	9	9
**% in clinical strains**	71	71	71	71

### Influence of Plasmid Presence on Strain Phenotypes

**Figure 2:**
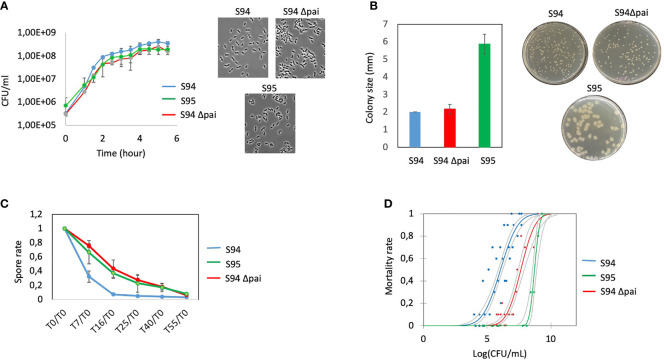
**(A)** left: Determination of growth curves of *B cereus* strains (S94, S95, S94Δpai) by measuring the OD_600nm_ in BHI medium at 37°C during 6.5 hours. Each point is the mean of four independent experiments. Vertical bars indicate standard errors. Right: representative images of microscopic observation of the strains at DO_600 nm_ = 0.3. **(B)** *B. cereus* strains) were plated on BHI plates to obtained single colonies. Left: the sizes of 25 colonies per plate were measured. The results indicate the mean of three independent experiments with standard errors; Right: representative images of the strains. **(C)** Germination rate efficiency measured over 55 minutes. The spores of the strains were incubated in BHI and at each time point, the remaining heat resistant bacteria (spores) were measured and normalized to the initial spore values at t0. The decrease in spore value indicate the rate of germination. The results indicate the mean of three independent experiments with standard errors. **(D)** Mortality rate of *G mellonella* injected with increasing doses of the strains was assessed 24 hours post-infection. Each dot represents the data for 10 larvae. Several dots are overlapping for the S95 strain.

The first and last isolates, S94 and S95, were evaluated in various phenotypical and functional assays. The two strains have similar growth capacity over time although the S95 strain was slightly impaired (**Figure 2A**). The two strains show no apparent difference in morphology. However, the colony morphology on plate is different with S95 colonies being wider (**Figure 2B**). In average, the size of the colonies was 3 times higher for the S95 strain compared to the S94 strain. In addition, S95 strain showed a drastic diminution in its germination rate compared to the S94 strain (**Figure 2C**). Finally, the S95 strain was severely impaired in its virulence potential compared to the S94 strain in the insect model of infection (**Figure 2D**). The lethal doses 50 (LD50) were 1,16.10^6^ CFU/mL for the S94 strain and 5,29.10^8^ CFU/mL for the S95 strain, thus displaying a 456-fold difference.

Taken together, these data strongly suggest that the plasmid pAH187_270 carried by the S94 strain and lost by the S95 strain plays an essential role during *B. cereus* virulence.

### Role of the PAI on Strain Phenotypes

To confirm that the pAH187_270 plasmid has an influence on the strain pathogenicity, we first tried to cure the S94 strain from this plasmid. However, all our attempts to cure the plasmid were unsuccessful. Thus, we focussed on the PAI region containing most of the markers previously identified as characteristic of clinical strains. To assess whether the genes located in the PAI may explain the difference in the strain pathogenicity, the S94 strain was deleted for its PAI and the Δpai-mutated strain was compared with the wild type strain in various phenotypical and functional assays.

The strains were observed under the microscope and bacterial morphology showed that the two strains were almost similar in cellular shape and size although the Δpai mutant was slightly impaired in its growth capacity (**Figures 2A, B**).

As at least two germination genes were identified within the PAI (**Figure 1**), the capacity of the wt and mutant strain to sporulate and germinate was assessed. No difference in the sporulation efficiency was observed (not shown). However, the Δpai mutant showed a drastic diminution in its germination rate (**Figure 2C**). The same difference was observed for the S95 and the Δpai mutant compared to the S94 strain, strongly suggesting that the PAI is responsible for the germination efficiency of the initial S94 strain.

Finally, to assess whether the genes located in the PAI may explain the difference in the strain pathogenicity, the S94 and the Δpai mutated strains were evaluated in the *Galleria* infection model (**Figure 2D**). Strikingly, the Δpai mutant was severely impaired in its virulence potential compared to the wild type strain. The lethal doses (LD) was 3.80E+07 CFU/mL for the Δpai mutant strain, thus displaying a 34.5 fold difference, indicating that the PAI plays an important role in *B. cereus* virulence capacity.

The S95 strain showed morphological difference to the Δpai mutant and was even more severely impaired in its virulence capacity. This implies that the genes located within the PAI play an important role during *B. cereus* virulence, but that other genes located elsewhere in the plasmid are also required.

## Discussion

Here, we report the identification of a large plasmid, lost over time within the same patient. This plasmid plays an essential role during *B. cereus* germination and virulence in an insect model. Notably, in strain S94, several biomarkers were found on the single large plasmid pAH187_270 within a newly identified pathogenicity island, providing further insights into *B. cereus* pathogenicity and complexity.

Within the *B. cereus sensu lato* group, the carriage of different virulence plasmids was traditionally considered a major contributor to the phenotypic properties that were critical for the speciation of *B. anthracis*, *B. cereus*, and *B. thuringiensis* (Rasko et al., 2007; Adams et al., 2014). However, recent findings suggest a need to reconsider traditional species assignments upon plasmid-mediated pathogenic phenotypes, in particular with the isolation of several *B. cereus* strains from patients with respiratory anthrax-like symptoms and carrying a pXO1-like plasmid (Baldwin, 2020). The identification of the pAH187_270 as an important plasmid for *B. cereus* pathogenicity paves the way for future investigation and may indicate that pAH187_270 could be a marker of clinically relevant *B. cereus* strains.

In particular, a previous study carried out on strains representative of *B. cereus* pathogenicity concluded that most clinical strains possess the combination of 4 genetic biomarkers, named *adhB*, *agrC*, *thiJ* and *BCQ_PI180* (Kavanaugh et al., 2021; Kavanaugh et al., 2022). Strikingly, the biomarker *BCQ_PI180* could be exchanged with other genes (ie: *gshAB, BCQ_PI181, ger(x)C*) giving similar results during the AUC analysis, with an AUC of 0.955 in all cases (Kavanaugh et al., 2021). We consistently showed here that these genes belong to the a PAI located on a large plasmid. The presence of these genes was assessed in a collection of clinical and non clinical strains and revealed that they were present in 25/35 (71%) clinical isolates and 2 of 21 (9%) of non clinical isolates, respectively (**Table 3**) (Kavanaugh et al., 2021), further highlighting their importance during *B. cereus* pathogenesis.

The presence of the *ces* gene is normally associated with emetic *B. cereus* strains, which constitute a cluster of food-borne outbreak (FBO) related strains and represent less than 1% of the strains identified during FBO. This is not surprising as the emetic syndrome is due to the ingestion of the cereulide, pre-formed in food, and does not necessarily require ingestion of the emetic strain itself. However, it has been previously shown that several *ces*-positive strains induced non-emetic symptoms (Glasset et al., 2016). This may be due to the fact that other genes, carried by the same plasmid, may have induced these non-emetic symptoms. Similarly, we have previously shown that strains carrying the *ces* gene were found to induce non-gastrointestinal clinical symptoms (Glasset et al., 2018). The markers present on this plasmid may have been at the origin of the symptoms, independently of the *ces* gene. The pAH187 plasmid presents a high degree of sequence similarity to the *B. anthracis* pXO1 plasmid (Rasko et al., 2007), but lacks the pXO1 pathogenicity island. By contrast, we have shown that this plasmid contains a specific pathogenicity island that defines pathogenic *B. cereus* isolates.

Several bacteria carry some of their virulence determinants on specific chromosome or plasmid locations referred to as pathogenicity islands (Parsot, 1994; Cossart and Lecuit, 1998). So far, a pathogenicity island had never been described for *B. cereus*. This island is approximately 50 kb and flanked by resolvases and transposases, likely indicating an acquisition or fusion event. The deletion of this region decreases *B. cereus* virulence in an insect model of infection. Together, it is tempting to speculate that this plasmid carrying the *B. cereus* pathogenic island may define clinical *B. cereus* in the same way that pXO1 and pXO2 define the *B. anthracis* species.

The new discovery of unknown factors located on this plasmid paves the way for future research on their exact roles during *B. cereus* virulence. These data provide new hints into the role of large plasmids and plasmid-hosted genes in the virulence within the *B. cereus* group.

The patient underwent a severe antibiotic treatment and finally recovered from the infection. As the three isolates from this patient were sensitive to the antibiotics used, the treatment might have been successful. On the other hand, it is tempting to speculate that the antibiotic pressure and/or the immune system might have led to a loss of the pAH187_270 plasmid and that the new isolate, cured of the plasmid was rendered less pathogenic and was thus cleared.

In addition, the last isolate showed an acquired resistance to rifampicin, although no rifampicin was given to the patient. Nevertheless, this strongly suggests that, similarly to *S. aureus*, rifampicin should be used with caution to treat *B. cereus* infections.

Taken together, these new findings help in the understanding of *B. cereus* pathogenic potential and complexity and provide further hints into the role of large plasmids in the virulence of *B. cereus* strains. This may provide tools for a better assessment of the risks associated with *B. cereus* hospital contamination to improve hygiene procedure and patient health.

## Data Availability Statement

The data presented in this study are deposited in the https://www.ebi.ac.uk/ena/ repository, accession number PRJEB46455.

## Ethics Statement

Ethical review and approval were not required for the study on human participants in accordance with the local legislation and institutional requirements. Written informed consent for participation was not required for this study in accordance with the national legislation and the institutional requirements.

## Author Contributions

DK, RD, DC, and BG: performed experiments and analyzed data. NR: supervision, analyzed data, writing of manuscript, and funding sources. All authors contributed to the article and approved the submitted version.

## Funding

This work was supported by the European EJP CARE project from the European Union’s Horizon 2020 research and innovation program under Grant Agreement No. 773830.

## Conflict of Interest

The authors declare that the research was conducted in the absence of any commercial or financial relationships that could be construed as a potential conflict of interest.

## Publisher’s Note

All claims expressed in this article are solely those of the authors and do not necessarily represent those of their affiliated organizations, or those of the publisher, the editors and the reviewers. Any product that may be evaluated in this article, or claim that may be made by its manufacturer, is not guaranteed or endorsed by the publisher.
